# Concurrent use of prescription gabapentinoids with opioids and risk for fall-related injury among older US Medicare beneficiaries with chronic noncancer pain: A population-based cohort study

**DOI:** 10.1371/journal.pmed.1003921

**Published:** 2022-03-01

**Authors:** Cheng Chen, Almut G. Winterstein, Wei-Hsuan Lo-Ciganic, Patrick J. Tighe, Yu-Jung Jenny Wei

**Affiliations:** 1 Department of Pharmaceutical Outcomes and Policy, University of Florida College of Pharmacy, Gainesville, Florida, United States of America; 2 Center for Drug Evaluation and Safety, University of Florida, Gainesville, Florida, United States of America; 3 Department of Epidemiology, University of Florida Colleges of Medicine and Public Health & Health Professions, Florida, United States of America; 4 Department of Anesthesiology, University of Florida College of Medicine, Florida, United States of America; Universite de Paris Faculte de Sante, FRANCE

## Abstract

**Background:**

Gabapentinoids are increasingly prescribed to manage chronic noncancer pain (CNCP) in older adults. When used concurrently with opioids, gabapentinoids may potentiate central nervous system (CNS) depression and increase the risks for fall. We aimed to investigate whether concurrent use of gabapentinoids with opioids compared with use of opioids alone is associated with an increased risk of fall-related injury among older adults with CNCP.

**Methods and findings:**

We conducted a population-based cohort study using a 5% national sample of Medicare beneficiaries in the United States between 2011 and 2018. Study sample consisted of fee-for-service (FFS) beneficiaries aged ≥65 years with CNCP diagnosis who initiated opioids. We identified concurrent users with gabapentinoids and opioids days’ supply overlapping for ≥1 day and designated first day of concurrency as the index date. We created 2 cohorts based on whether concurrent users initiated gabapentinoids on the day of opioid initiation (Cohort 1) or after opioid initiation (Cohort 2). Each concurrent user was matched to up to 4 opioid-only users on opioid initiation date and index date using risk set sampling. We followed patients from index date to first fall-related injury event ascertained using a validated claims-based algorithm, treatment discontinuation or switching, death, Medicare disenrollment, hospitalization or nursing home admission, or end of study, whichever occurred first. In each cohort, we used propensity score (PS) weighted Cox models to estimate the adjusted hazard ratios (aHRs) with 95% confidence intervals (CIs) of fall-related injury, adjusting for year of the index date, sociodemographics, types of chronic pain, comorbidities, frailty, polypharmacy, healthcare utilization, use of nonopioid medications, and opioid use on and before the index date. We identified 6,733 concurrent users and 27,092 matched opioid-only users in Cohort 1 and 5,709 concurrent users and 22,388 matched opioid-only users in Cohort 2. The incidence rate of fall-related injury was 24.5 per 100 person-years during follow-up (median, 9 days; interquartile range [IQR], 5 to 18 days) in Cohort 1 and was 18.0 per 100 person-years during follow-up (median, 9 days; IQR, 4 to 22 days) in Cohort 2. Concurrent users had similar risk of fall-related injury as opioid-only users in Cohort 1(aHR = 0.97, 95% CI 0.71 to 1.34, *p* = 0.874), but had higher risk for fall-related injury than opioid-only users in Cohort 2 (aHR = 1.69, 95% CI 1.17 to 2.44, *p* = 0.005). Limitations of this study included confounding due to unmeasured factors, unavailable information on gabapentinoids’ indication, potential misclassification, and limited generalizability beyond older adults insured by Medicare FFS program.

**Conclusions:**

In this sample of older Medicare beneficiaries with CNCP, initiating gabapentinoids and opioids simultaneously compared with initiating opioids only was not significantly associated with risk for fall-related injury. However, addition of gabapentinoids to an existing opioid regimen was associated with increased risks for fall. Mechanisms for the observed excess risk, whether pharmacological or because of channeling of combination therapy to high-risk patients, require further investigation. Clinicians should consider the risk–benefit of combination therapy when prescribing gabapentinoids concurrently with opioids.

## Abstract 2

In a cohort study, Cheng Chen and colleagues investigate associations between concurrent use of gabapentinoids and opioids and risk of fall-related injury, compared with use of opioids alone, among adults aged 65 years or older with chronic noncancer pain in the United States.

## Author summary

**Why was this study done?:**

- Prescriptions for gabapentinoids, commonly coadministered with opioids to manage chronic pain, have tripled among older adults in the past decade.
- Concurrent use of prescription opioids and gabapentinoids may potentiate central nervous system (CNS) depression and result in falls and related injuries, but this association has not been formally investigated.

**What did the researchers do and find?:**

- We conducted a population-based cohort study among older Medicare beneficiaries with chronic noncancer pain (CNCP) who initiated prescription opioids.
- We assembled 2 cohorts from opioid initiators: Cohort 1 included 6,733 patients initiating opioids and gabapentinoids simultaneously and 27,092 matched controls initiating opioids only, and Cohort 2 included 5,709 patients initiating gabapentinoids after opioid initiation and 22,388 matched controls continuing with opioids only.
- We found that concurrent gabapentinoid–opioid use was not associated with risk of fall-related injury in Cohort 1 but was associated with a 69% higher risk of fall-related injury in Cohort 2, when compared with use of opioid only.

**What do these findings mean?:**

- In this sample of older Medicare beneficiaries with CNCP, initiating gabapentinoids and opioids simultaneously compared with initiating opioids only was not associated with risks for fall-related injury.
- However, addition of gabapentinoids to an existing opioid regimen was associated with increased risks for fall-related injury.
- Clinicians should consider the risk–benefit of combination therapy when prescribing gabapentinoids concurrently with opioids.

## Introduction

Amid the opioid epidemic in the US, the proportion of older adults with gabapentinoid use (i.e., gabapentin and pregabalin) has more than tripled from 2.6% in 2002 to 8.4% in 2015 [1]. Between 2003 and 2016, more than one-third of physician office visits with gabapentinoid prescriptions involved an overlapping opioid prescription among older patients [2]. This prevalent coprescribing of opioids and gabapentinoids was largely attributable to the demonstrated clinical benefits of combining opioids with gabapentinoids on pain control [3,4]. In clinical trials, pain severity was significantly reduced after gabapentinoid treatment among patients who were new [4–6] or prevalent opioid users [7–9]. Both the 2009 American Geriatric Society (AGS) guideline and the 2016 Center for Disease Control and Prevention (CDC)’s guidance on opioid prescribing recommended the use of adjuvant analgesics including gabapentinoids, alone or in combination with opioids to reduce consumption of opioids and their associated adverse events for all age groups of patients with chronic noncancer pain (CNCP), including older adults [10,11].

There have been, however, growing safety concerns over the concurrent use of prescription opioids and gabapentinoids because both drugs can depress the central nervous system (CNS) [12–14]. Animal studies have shown coadministration of gabapentinoids and opioids may potentiate the respiratory depressing effect of opioids [15]. Recent observational studies among adults aged 18 years or older have reported that concurrent use was associated with increased risks for opioid overdose and respiratory complications, compared to those using opioids alone [16–19]. Considering their pharmacodynamic properties, concurrent use of gabapentinoids and opioids may also cause oversedation, resulting in falls and related injuries [14]—a severe health concern prevailing among older adults [20]. Yet, little evidence exists to quantify these fall risks, which are essential to inform decisions about prescribing gabapentinoids to older patients treated with opioids.

The objective of the present study is to examine the association between concurrent use of prescription gabapentinoids with opioids versus opioids alone and the risk for fall-related injury in older adults with CNCP using US Medicare claims data. To mitigate bias arising from observed (e.g., clinical comorbidities) and unobserved (e.g., duration and severity of pain conditions) differences between new and prevalent opioid users [21], we examined the association in 2 separate cohorts: older adults (1) who were new opioid users and simultaneously initiated gabapentinoids; and (2) who were prevalent opioid users and had gabapentinoids added to the existing opioid therapy. In both cohorts, we hypothesized that concurrent use of prescription gabapentinoids and opioids was associated with an increased risk for fall-related injury compared with prescription opioids only.

## Methods

### Study design and data source

We conducted a retrospective cohort study using a 5% national sample of Medicare beneficiaries from 2011 to 2018. Medicare is the largest health insurance provider for US older adults, covering 98% of this population [22]. The Medicare claims database contains billing records for inpatient (Part A) stays, outpatient (Part B) encounters, and dispensed prescription drugs covered under Medicare Part D. Medicare data also provide beneficiary level information on sociodemographic characteristics and monthly enrollment status. The study protocol was prespecified (S1 Text) with approval from the University of Florida Institutional Review Board (approval number IRB202001057), and the reporting of this study followed the Strengthening the Reporting of Observational Studies in Epidemiology (STROBE) statement (S1 STROBE Checklist).

### Study sample

We constructed our study sample in 2 steps by first assembling a source population of adults aged 65 or older who were newly prescribed opioids between January 1, 2011 and December 31, 2018 and then selecting concurrent gabapentinoid–opioid users (exposed group) and opioid-only users (unexposed group) from the source population of opioid initiators. Opioid initiators were defined as beneficiaries without prescription opioids in the 6 months before filling a prescription for opioids. During the 6 months prior to opioid initiation, patients were required to have (1) no gabapentinoid use; (2) continuous enrollment in Medicare Parts A, B, and D without insurance coverage from Medicare advantage plans; and (3) a diagnosis of a CNCP condition (S1 Table). We excluded beneficiaries who had a cancer diagnosis, hospice care, or palliative care, as well as those with a history of fall-related injury in the 6 months before or on the opioid initiation date.

### Identification of concurrent gabapentinoid–opioid users and opioid-only users

**Fig 1:**
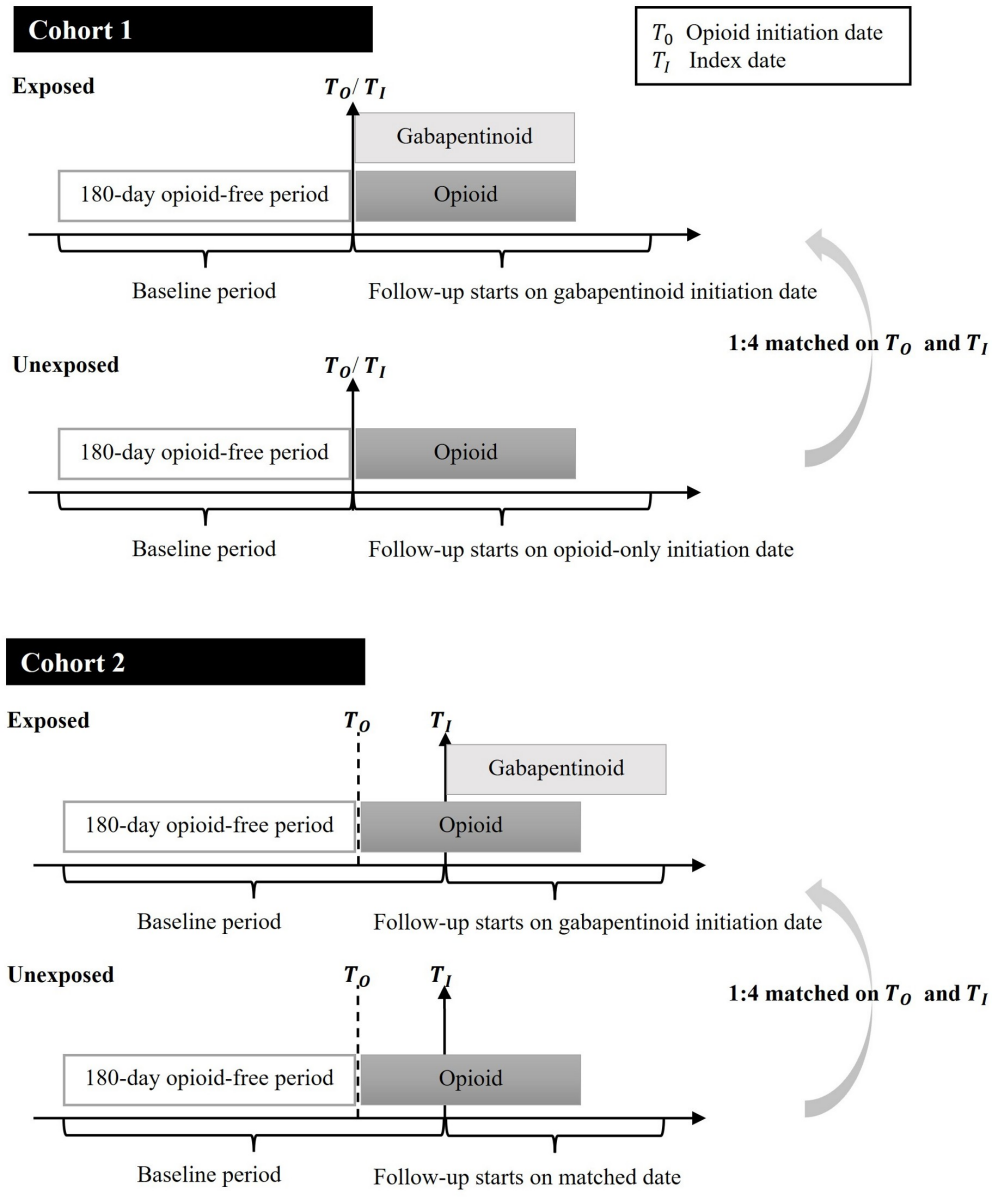
Description of 2 study cohorts of older adults with CNCP. The exposed group consists of concurrent gabapentinoid–opioid users, and the unexposed group consists of opioid-only users. T_0_ indicates date of opioid initiation, i.e., date of first opioid prescription after a 180-day opioid-free period. T_I_ indicates the index date, i.e., the beginning of follow-up. For the exposed group, T_I_ is the first day of concurrency. In Cohort 1, T_0_ and T_I_ overlap. In Cohort 2, T_0_ precedes T_I_. In both Cohort 1 and Cohort 2, each exposed patient is matched to up to 4 unexposed patients on T_0_ and T_I_. CNCP, chronic noncancer pain.

Among the eligible opioid initiators, we next identified concurrent users who had gabapentinoids and opioids days’ supply overlapping for at least 1 day and designated the first day of concurrent use as the index date. We categorized concurrent users into 2 groups and formed cohorts, respectively: (1) Cohort 1: concurrent users who initiated gabapentinoids and opioids simultaneously on the same date; and (2) Cohort 2: concurrent users who initiated gabapentinoids after opioid initiation (Fig 1). In both cohorts, each concurrent user was matched to up to 4 random opioid-only users based on opioid initiation date. Opioid-only users were assigned the same index date as the concurrent user they were matched to. This sampling approach allows for efficiently selecting opioid-only users that represent the underlying unexposed population [23]. Patients were excluded if they had a hospital or Part A skilled nursing facility (SNF) stay that extended over the index date, during which Part D prescription data were incomplete [24]. In Cohort 2, between the opioid initiation and the index date, we further required patients to be on prescription opioids without any treatment gaps of 1 day or more and be free of cancer diagnosis, hospice or palliative care, or fall-related injury.

### Exposure

We measured gabapentinoid and opioid exposure based on the dispensing date and days’ supply of prescription fills reimbursed by Medicare Part D plans. To account for delays in prescription fills, we extended the days’ supply for each prescription by 20% [25].

### Outcome and follow-up

Our outcome of interest was the first medical encounter with a diagnosis or procedure code indicative of a fall-related injury. We used a previously developed algorithm that includes the International Classification of Diseases, Ninth/Tenth Revision, Clinical Modification, and Current Procedural Terminology codes for fall-related injuries [26]. The algorithm has been validated against a composite standard of an external cause of injury from claims or confirmation by respondents in a US national survey, with a sensitivity of 62.1%, a specificity of 98.8%, and a positive predictive value of 88.6% [26].

Follow-up started on the index date and ended with the first of the following events: incident fall-related injury, discontinuation of opioids, discontinuation of gabapentinoids (exclusively for concurrent users), initiation of gabapentinoids (exclusively for opioid-only users), death, end of enrollment in Medicare fee-for-service (FFS) programs, admission to either hospital or SNF, day 30 after the index date, or December 31, 2018.

### Covariates

Covariates were measured during a baseline period, defined for Cohort 1 as 6 months before opioid initiation, and for Cohort 2 as 6 months before opioid initiation plus the days between opioid initiation and index date to account for preindex opioid use (Fig 1). Covariates considered in the study (S2 Table) included calendar year of the index date, sociodemographics (age, sex, race/ethnicity, metropolitan residency, low-income subsidy status, and census region classified based on the US census bureau grouping of states), types of chronic pain condition (back pain, neck pain, osteoarthritis, rheumatoid disease, gout, other joint pain, other musculoskeletal pain, neuropathy or neuralgia, fibromyalgia, abdominal pain, migraine, and unspecified chronic pain), other comorbidities, frailty measured using a validated claims-based index [27], polypharmacy (i.e., use of 5 or more different medications), healthcare utilization (any inpatient stays, any emergency department [ED] visits, any use of SNF services, and any bone mineral density testing as a proxy for bone fragility), use of other medications associated with fall risks, and opioid measures on the index date. For medication use associated with falls [14,21], we considered use of antihistamines, angiotensin converting enzyme inhibitors, angiotensin II receptor antagonists, thiazide diuretics, calcium channel blockers, beta-blockers, loop diuretics, antiosteoporosis medications, oral steroids, muscle relaxants, and CNS-active drugs including anticonvulsants (except gabapentinoids), tricyclic antidepressants, selective serotonin reuptake inhibitors, serotonin and norepinephrine reuptake inhibitors, antipsychotics, benzodiazepines, and nonbenzodiazepine sedatives/hypnotics. As opioid use alone may increase the risk for fall-related injury, we also accounted for opioid daily dose in morphine milligram equivalents (MMEs) and use of long-acting opioids (yes/no) (see the list of long-acting opioids in S3 Table) on the index date. For Cohort 2, we further considered opioid use duration, average daily MME, maximum daily MME, and use of long-acting opioid (yes/no) between opioid initiation date and index date.

### Statistical analysis

We conducted statistical analyses separately for the Cohort 1 and Cohort 2. In each cohort, we adjusted for baseline covariates via inverse probability of treatment weights (IPTWs)—the recommended propensity score (PS) method for estimating the average treatment effect in the overall population [28]. In IPTW, the exposed group (i.e., concurrent users) received weights of the inverse of the estimated PS, whereas the unexposed group (i.e., opioid-only users) received weights equal to the inverse of 1 minus the estimated PS. We estimated the PS score of a patient receiving concurrent use versus opioid only given the set of baseline covariates by using logistic regression. To increase precision of final association estimates, we further used stabilized IPTW method in which the weight was multiplied by the marginal probability of receiving the corresponding treatment (i.e., concurrent therapy for exposed and opioid monotherapy for unexposed) [29]. To minimize the influence of outliers on the final estimates, we trimmed weights outside the first and 99th percentile. We assessed covariates balance using standardized mean differences (SMDs), wherein an SMD >0.10 indicates imbalance. In each IPTW-weighted cohort, to illustrate the risks of fall-related injury over time, we plotted Kaplan–Meier survival curves adjusting for baseline covariates. To quantify the association between concurrent therapy and fall-related injury, we used IPTW-weighted Cox proportional hazard models with robust estimation to calculate adjusted hazard ratios (aHRs) and 95% confidence intervals (CIs). We used Schoenfeld residuals to test the proportional hazard assumption for the key exposure of interest and detected no violation [30]. These analyses were prospectively planned in the study protocol (S1 Text).

To evaluate the robustness of our main findings, we conducted 3 prespecified sensitivity analyses. First, to address concerns about informative censoring, we conducted an intention-to-treat analysis where patients were considered remaining in the initial treatment group, regardless of discontinuation or switching during follow-up. Second, to account for changes in comedication use during follow-up that may influence fall risks, we modeled daily MME, use of long-acting opioids, and use of other medications associated with fall risks as time-varying covariates. Third, to reduce potential bias arising from prior fractures or osteoporosis, which are strong predictors for subsequent fall-related injury [31] and may explain gabapentinoid use [32,33], we excluded patients who had a diagnosis of these 2 conditions or had antiosteoporosis prescriptions filled during the baseline period.

To assess whether the association varied by treatment patterns, we conducted 3 prespecified subgroup analyses in each cohort according to individual gabapentinoid agents (i.e., gabapentin or pregabalin), duration of concurrent use, and starting dose of gabapentinoid treatment. We considered high starting dose as >300 mg/day for gabapentin or >150 mg/day for pregabalin, the recommended starting dose per FDA-approved label [34,35]. We also stratified the association by age group (dichotomized as 65 to 74 and ≥75), sex, use of benzodiazepines at baseline (yes/no), and prescription opioid dose (dichotomized as <20 MME and ≥20 MME) on the index date to understand whether these patient characteristics modify the association between concurrent gabapentinoid–opioid use and risk for fall-related injury.

For all sensitivity and subgroup analyses in which a subset of samples was used, we recalculated the IPTWs and reassessed the balance of the covariates. All tests were set at α = 0.05 level, 2-sided. We used SAS 9.4 (SAS institute, Cary, North Carolina, US) to analyze the data.

## Results

### Patients’ characteristics

**Fig 2:**
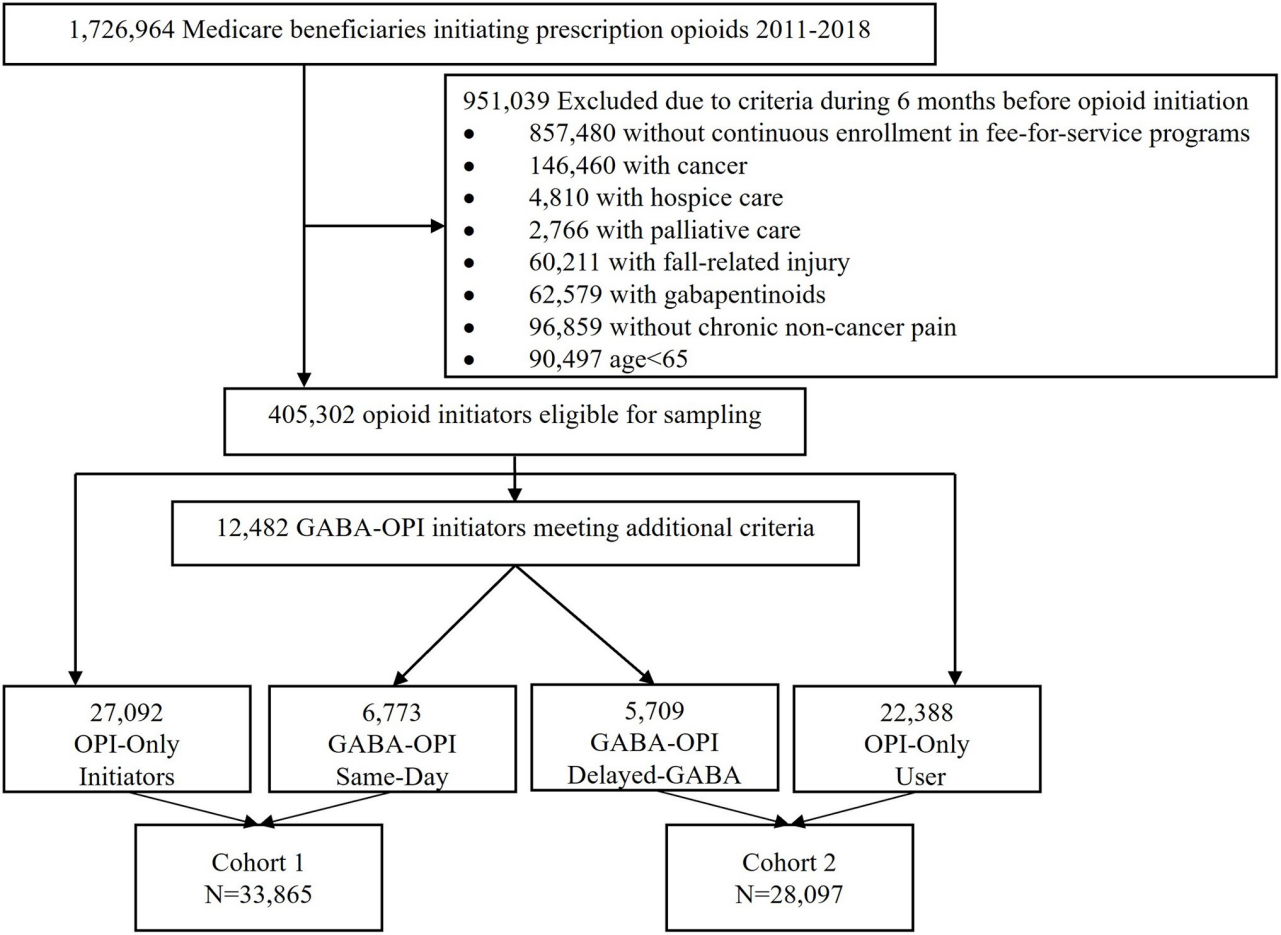
Flowchart of sample selection. GABA, gabapentinoid; OPI, opioid.

In Cohort 1, we identified 6,773 eligible older adults who initiated gabapentinoids and opioids simultaneously and sampled 27,092 opioid-only initiators as the reference. In Cohort 2, we identified 5,709 eligible older adults who initiated gabapentinoids with existing opioid use after opioid initiation and sampled 22,388 patients who continued with an opioid-only regimen as the reference (Fig 2). Table 1 summarizes the selected characteristics of eligible patients in each cohort before and after IPTW weighting (a complete list of covariates in S2 Table). In both cohorts, concurrent users had a higher prevalence of back pain, neck pain, neuralgia, and unspecified chronic pain than opioid-only users. On the index date, concurrent users were more likely to receive opioid doses ≥90 MME and long-acting opioids compared with opioid-only users. After incorporating IPTW and weight trimming, all baseline characteristics were balanced in both cohorts.

**Table 1:** Selected baseline characteristics of older adults with CNCP by treatment with concurrent use of gabapentinoids and opioids or opioids only.

Characteristics	Cohort 1, %				Cohort 2, %			
	Unweighted		Weighted		Unweighted		Weighted	
	Gabapentinoids with opioids (*n* = 6,733)	Opioids only (*n* = 27,092)	Gabapentinoids with opioids (*n* = 6,349)	Opioids only (*n* = 27,156)	Gabapentinoids with opioids (*n* = 5,709)	Opioids only (*n* = 22,388)	Gabapentinoids with opioids (*n* = 5,475)	Opioids only (*n* = 22,322)
Age, mean ± SD, y	75.0 ± 7.0	75.7 ± 7.6	75.4 ± 7.0	75.6 ± 7.5	75.7 ± 7.3	76.5 ± 7.8	76.3 ± 7.4	76.4 ± 7.8
Female	66.0	64.1	65.4	64.5	67.9	67.5	68.1	67.6
Race/ethnicity								
Non-Hispanic White	77.4	81.8	80.2	80.9	80.7	79.2	79.6	79.5
Black	8.1	8.0	8.2	8.0	7.0	9.1	8.4	8.7
Hispanic	8.2	5.9	6.6	6.3	7.2	7.1	7.4	7.1
Asian/Pacific Islander	4.2	2.3	3.0	2.7	3.3	2.8	3.0	2.9
Others	2.1	2.0	2.0	2.0	1.7	1.8	1.6	1.8
Low-income subsidy	26.0	22.7	24.4	23.4	25.0	28.6	28.5	27.9
US region^a^								
Northeast	16.3	16.5	15.7	16.3	17.3	15.7	16.3	16.1
Northcentral	22.2	23.5	23.1	23.2	22.0	23.5	22.3	23.2
South	42.5	41.6	42.6	41.9	41.5	42.3	42.3	42.2
West	19.0	18.4	18.6	18.6	19.2	18.4	19.2	18.6
Chronic pain								
Back pain	47.1	36.1	41.6	38.5	62.1	42.9	50.5	46.8
Neck pain	16.8	12.4	14.9	13.4	20.7	13.8	17.2	15.3
Osteoarthritis	48.1	41.4	44.5	42.8	42.0	47.5	46.4	46.5
Neuropathy	53.7	25.7	34.9	31.6	66.2	28.7	38.9	36.2
Unspecified	12.4	7.6	9.6	8.6	16.3	10.1	13.2	11.5
Comorbidities								
Diabetes	36.0	31.0	33.9	32.1	36.4	33.9	35.8	34.6
Fracture	4.2	5.5	5.2	5.2	6.4	6.3	6.6	6.4
Osteoporosis	11.3	10.2	10.7	10.4	13.2	12.5	13.7	12.7
ADRD	4.1	5.5	5.0	5.2	5.9	7.1	7.0	6.9
Alcohol/tobacco use	9.0	7.8	8.1	8.0	10.8	8.4	9.6	8.9
OUD	0.9	0.3	0.5	0.4	1.8	0.6	1.0	0.8
Frailty index, mean ± SD	0.2 ± 0.1	0.2 ± 0.1	0.2 ± 0.1	0.2 ± 0.1	0.2 ± 0.1	0.2 ± 0.1	0.2 ± 0.1	0.2 ± 0.1
Healthcare utilization								
Polypharmacy	92.2	84.3	88.3	85.9	83.3	79.3	81.6	80.1
Any hospitalization	30.8	17.5	22.0	20.4	20.3	19.0	20.6	19.3
Any ED visit	26.3	28.8	27.8	28.3	39.0	27.2	30.4	29.4
Any SNF services	5.3	2.9	3.8	3.4	4.7	4.1	4.6	4.3
Baseline medication use								
Anticonvulsants	2.6	3.5	3.0	3.3	3.7	3.9	4.2	3.9
TCAs	3.3	2.6	2.9	2.7	3.5	3.2	3.4	3.2
SNRIs	4.4	4.4	4.8	4.4	6.3	5.0	5.9	5.4
SSRIs	16.2	16.4	17.0	16.4	18.4	17.8	18.4	17.9
Antipsychotics	2.3	3.4	3.0	3.2	4.1	4.5	4.9	4.5
Benzodiazepines	12.6	13.8	13.7	13.6	16.9	15.3	15.9	15.7
Nonbenzodiazepines^b^	6.8	6.3	6.5	6.4	7.1	6.8	6.8	6.8
Muscle relaxants	12.8	9.3	11.1	10.1	18.7	11.0	13.3	12.5
Antiosteoporosis	5.8	5.4	5.5	5.4	7.1	6.5	6.6	6.5
Oral steroids	30.1	27.4	29.3	28.0	42.9	27.8	32.2	30.8
Index opioid prescription								
MME group								
<20	33.6	28.9	30.1	29.7	34.6	45.4	43.3	43.4
20 to 49	46.1	55.8	53.2	53.8	45.2	44.0	43.4	44.3
50 to 89	13.0	12.2	12.2	12.4	13.5	7.7	9.1	8.7
≥90	7.3	3.1	4.5	4.1	6.7	2.9	4.2	3.6
Long-acting	2.3	0.7	1.2	1.0	3.6	2.0	3.1	2.4
Baseline opioid use								
Days, mean ± SD	–	–	–	–	17.8 ± 34.5	15.2 ± 24.5	16.6 ± 29.7	15.8 ± 27.5
Average MME								
<20	–	–	–	–	41.2	46.5	44.6	45.4
20 to 49	–	–	–	–	48.0	43.6	44.5	44.5
50 to 89	–	–	–	–	8.3	7.5	8.1	7.7
≥90	–	–	–	–	2.4	2.3	2.8	2.4
Max MME								
<20	–	–	–	–	35.6	42.3	39.7	40.9
20 to 49	–	–	–	–	45.5	44.8	44.6	44.9
50 to 89	–	–	–	–	13.1	9.0	10.6	9.9
≥90					5.8	3.9	5.1	4.3
Long-acting	–	–	–	–	3.4	2.0	3.1	2.4

Data are percent unless otherwise indicated.

^a^US region was defined by the state code of a beneficiary’s residence and classified per the US census bureau.

^b^Nonbenzodiazepines included eszopiclone, zaleplon, and zolpidem.

ADRD, Alzheimer disease and related dementia; ED, emergency department; MME, morphine milligram equivalent; OUD, opioid use disorder; SD, standard deviation; SNF, skilled nursing facility; SNRI, serotonin and norepinephrine reuptake inhibitor; SSRI, selective serotonin reuptake inhibitor; TCA, tricyclic antidepressant.

Comparing Cohorts 1 and 2, we noticed that Cohort 2 had higher proportions of patients with back pain, neck pain, neuralgia, unspecified chronic pain, opioid use disorder (OUD), use of muscle relaxants and use of benzodiazepines at baseline, and use of long-acting opioids on the index date. Before weighting, the SMDs of the proportions of patients with OUD, ED visits, use of muscle relaxants, and use of oral steroids were larger in Cohort 2 than in Cohort 1(S4 Table), indicating that the differences of these characteristics between the 2 groups were more substantial in Cohort 2 than those in Cohort 1.

### Main results

In Cohort 1, 279 beneficiaries experienced a fall-related injury with an incidence rate of 24.5 per 100 person-years (median follow-up time: 9 days; interquartile range [IQR] = 5 to 18). The risk of an incident fall-related injury did not differ between patients initiating gabapentinoids and opioids simultaneously versus those initiating opioids only (aHR = 0.97, 95% CI 0.71 to 1.34, *p* = 0.874), adjusting for index year, sociodemographics, chronic pain types, comorbidities, frailty, polypharmacy, healthcare utilization, and use of nonopioid medication during baseline as well as opioid measures (including dose and type) on the index date. The unadjusted and adjusted Kaplan–Meier curves both showed that the fall risks were similar in concurrent users and opioid-only users (S1 Fig).

In Cohort 2, 179 beneficiaries experienced a fall-related injury with an incidence rate of 18.0 per 100 person-years (median follow-up time: 9 days, IQR = 4 to 22) (Table 2). An increased risk of an incident fall-related injury was observed among patients initiating gabapentinoids with existing opioids compared with those continuing opioids only (aHR = 1.69, 95% CI 1.17 to 2.44, *p* = 0.005), adjusting for the covariates considered in Cohort 1 plus opioid measures (including dose, type, and duration) during baseline. The unadjusted and adjusted Kaplan–Meier curve both showed higher fall risks in concurrent users than in opioid-only users, particularly during the early days of the therapy (S2 Fig).

**Table 2:** Risk of fall-related injury comparing concurrent gabapentinoid–opioid users and opioid-only users.

Main results	Cohort 1			Cohort 2		
	Overall	Gabapentinoids with opioids	Opioids only	Overall	Gabapentinoids with opioids	Opioids only
# of patients	33,586	6,773	27,092	28,097	5,709	22,388
# of events	279	64	215	179	59	120
Median (IQR) follow-up, days	9 (5 to 18)	8 (5 to 16)	12 (6 to 26)	9 (4 to 22)	11 (5 to 25)	9 (4 to 22)
Incidence rate, per 100 person-years	24.5	22.2	25.3	18.0	26.6	15.6
Crude HR (95% CI), *p*-value	–	0.92 (0.70 to 1.22), *p* = 0.581	Reference	–	1.72 (1.26 to 2.35), *p* = 0.001	Reference
aHR^a^ (95% CI), *p*-value	–	0.97 (0.71 to 1.34), *p* = 0.874	Reference	–	1.69 (1.17 to 2.44), *p* = 0.005	Reference

^a^Both Cohort 1 and Cohort 2 adjusted for calendar year of the index date, sociodemographics, types of chronic pain, other comorbidities, frailty index, polypharmacy, healthcare utilization, and use of nonopioids during baseline as well as opioid measures (including dose and type) on the index date. Cohort 2 also adjusted for the opioid measures (including dose, type, and duration) during baseline.

aHR, adjusted hazard ratio; CI, confidence interval; HR, hazard ratio; IQR, interquartile range.

### Sensitivity analyses

All sensitivity analyses produced results consistent with the primary analyses (Table 3). In Cohort 1, we found no differential fall risks between concurrent users and opioid-only users, with the aHRs ranging from 0.96 (95% CI 0.72 to 1.29, *p* = 0.798) when modeling selected covariates in a time-varying manner to 1.07 (95% CI 0.73 to 1.57, *p* = 0.744) when excluding patients with baseline fracture or osteoporosis. In Cohort 2, we found increased fall risks in concurrent users versus opioid-only users, with the aHRs ranging from 1.34 (95% CI 1.00 to 1.78, *p* = 0.047) in intention-to-treat analysis to 1.90 (95% CI 1.23 to 2.95, *p* = 0.004) when excluding patients with baseline fracture or osteoporosis.

**Table 3:** Risk of fall-related injury comparing concurrent gabapentinoid–opioid users and opioid-only users in sensitivity analyses.

Sensitivity analysis	Cohort 1		Cohort 2	
	Gabapentinoids with opioids	Opioids only	Gabapentinoids with opioids	Opioids only
*Time-varying analysis*				
# of patients	6,773	27,092	5,709	22,388
# of events	64	215	59	120
Unadjusted HR (95% CI), *p*-value	0.92 (0.70 to 1.22), *p* = 0.581	Reference	1.72 (1.26 to 2.35), *p* = 0.001	Reference
aHR^a^ (95% CI), *p*-value	0.96 (0.72 to 1.29), *p* = 0.798	Reference	1.67 (1.22 to 2.29), *p* = 0.001	Reference
*Intention-to-treat analysis*				
# of patients	6,773	27,092	5,709	22,388
# of events	93	395	87	260
Unadjusted HR (95% CI), *p*-value	0.96 (0.76 to 1.20), *p* = 0.692	Reference	1.33 (1.05 to 1.70), *p* = 0.021	Reference
aHR^b^(95% CI), *p*-value	0.96 (0.74 to 1.25), *p* = 0.764	Reference	1.34 (1.00 to 1.78), *p* = 0.047	Reference
*No prior fracture or osteoporosis diagnosis*				
# of patients	5,610	22,472	4,537	17,978
# of events	47	132	43	82
Unadjusted HR (95% CI), *p*-value	1.09 (0.78 to 1.52), *p* = 0.632	Reference	1.84 (1.27 to 2.66), *p* = 0.001	Reference
aHR^b^ (95% CI), *p*-value	1.07 (0.73 to 1.57), *p* = 0.744	Reference	1.90 (1.23 to 2.95), *p* = 0.004	Reference

^a^Both Cohort 1 and Cohort 2 adjusted for calendar year of the index date, sociodemographics, types of chronic pain, other comorbidities, frailty index, polypharmacy, healthcare utilization, and use of nonopioids during baseline as well as opioid measures (including dose and type) on the index date. Cohort 2 also adjusted for the opioid measures (including dose, type, and duration) during baseline.

^b^Both Cohort 1 and Cohort 2 adjusted for all variables mentioned in footnote a plus time-varying covariates (including daily MME, use of long-acting opioids, and use of other medications associated with fall risks) during follow-up. Cohort 2 also adjusted for the opioid measures (including dose, type, and duration) during baseline.

CI, confidence interval; HR, hazard ratio; MME, morphine milligram equivalent.

### Subgroup analyses

Table 4 shows the results of subgroup analyses. In Cohort 1, no association was noticed between concurrent use and risk for fall-related injury across all subgroup analyses. The aHRs ranged from 0.53 (95% CI 0.25 to 1.12, *p* = 0.096) in males to 1.19 (95% CI 0.57 to 2.45, *p* = 0.644) in pregabalin-opioid users. In Cohort 2, we observed an increased fall risk within the first 1 to 14 days of concurrent use (aHR = 2.02, 95% CI 1.41 to 2.92, *p* < 0.001) but did not in the 15 to 30 days of concurrent use (aHR = 1.05, 95% CI 0.56 to 1.99, *p* = 0.870). When stratified by gabapentinoid medication, we saw an increased risk in gabapentin-opioid users (aHR = 1.82, 95% CI 1.26 to 2.64, *p* = 0.001), whereas not in pregabalin-opioid users (aHR = 1.22, 95% CI 0.45 to 3.31, *p* = 0.692), likely due to smaller sample size of this subgroup. We observed consistently elevated risks among high-dose gabapentinoid initiators (aHR = 1.90, 95% CI 1.13 to 3.19, *p* = 0.016) and low-dose gabapentinoid initiators (aHR = 1.84, 95% CI 1.16 to 2.94, *p* = 0.010), versus opioid-only users. In Cohort 2, we also observed concurrent use to be associated with increased fall risks among patients who were aged ≥75 (aHR = 1.59, 95% CI 1.03 to 2.46, *p* = 0.036), had no baseline use of benzodiazepine (aHR = 1.84, 95% CI 1.22 to 2.78, *p* = 0.004), and received opioid dose of ≥20 MME/day on the index date (aHR = 1.93, 95% CI 1.22 to 3.06, *p* = 0.005).

**Table 4:** Risk of fall-related injury comparing concurrent gabapentinoid–opioid users and opioid-only users among subgroups.

Subgroup analysis	Cohort 1				Cohort 2			
	Unadjusted HR (95% CI)	*p*-Value	aHR (95% CI)	*p*-Value	Unadjusted HR (95% CI)	*p*-Value	aHR (95% CI)	*p*-Value
*By treatment duration* ^a^								
0 to 14 days	0.95 (0.70 to 1.29)	0.726	1.03 (0.76 to 1.41)	0.842	1.96 (1.36 to 2.82)	<0.001	2.02 (1.41 to 2.92)	<0.001
15 to 30 days	0.82 (0.41 to 1.63)	0.575	0.71 (0.33 to 1.53)	0.384	1.22 (0.66 to 2.25)	0.530	1.05 (0.56 to 1.99)	0.870
*By individual agent* ^a^								
Gabapentin	0.86 (0.64 to 1.16)	0.334	0.93 (0.66 to 1.31)	0.679	1.78 (1.30 to 2.45)	<0.001	1.82 (1.26 to 2.64)	0.001
Pregabalin	1.26 (0.65 to 2.47)	0.499	1.19 (0.57 to 2.45)	0.644	1.38 (0.56 to 3.39)	0.485	1.22 (0.45 to 3.31)	0.692
*By gabapentinoid dose* ^a^								
Low dose	0.88 (0.61 to 1.26)	0.504	0.84 (0.54 to 1.30)	0.429	1.73 (1.17 to 2.57)	0.007	1.84 (1.16 to 2.94)	0.010
High dose	0.84 (0.54 to 1.30)	0.429	1.18 (0.76 to 1.83)	0.467	1.70 (1.13 to 2.57)	0.011	1.90 (1.13 to 3.19)	0.016
*By age group* ^b^								
65 to 74	1.13 (0.71 to 1.82)	0.608	1.12 (0.66 to 1.92)	0.669	2.49 (1.40 to 4.44)	0.002	1.78 (0.93 to 3.42)	0.083
≥75	0.88 (0.62 to 1.24)	0.461	0.90 (0.59 to 1.35)	0.596	1.58 (1.09 to 2.31)	0.017	1.59 (1.03 to 2.46)	0.036
*By sex* ^c^								
Male	0.64 (0.35 to 1.18)	0.152	0.53 (0.25 to 1.12)	0.096	2.02 (1.11 to 3.66)	0.021	2.15 (1.05 to 4.40)	0.036
Female	1.04 (0.75 to 1.43)	0.831	1.16 (0.81 to 1.65)	0.431	1.63 (1.13 to 2.36)	0.010	1.55 (1.01 to 2.37)	0.043
*By benzodiazepine use at baseline* ^d^								
Use	0.99 (0.47 to 2.08)	0.983	0.85 (0.35 to 2.05)	0.716	1.37 (0.67 to 2.78)	0.387	1.10 (0.51 to 2.36)	0.996
No use	0.91 (0.68 to 1.24)	0.564	1.01 (0.71 to 1.42)	0.973	1.81 (1.28 to 2.57)	0.001	1.84 (1.22 to 2.78)	0.004
*By index opioid dose* ^e^								
<20 MME	0.79 (0.51 to 1.22)	0.293	0.88 (0.53 to 1.44)	0.604	1.66 (0.99 to 2.79)	0.054	1.30 (0.71 to 2.37)	0.392
≥20 MME	1.06 (0.73 to 1.53)	0.778	1.13 (0.74 to 1.73)	0.584	1.61 (1.08 to 2.40)	0.021	1.93 (1.22 to 3.06)	0.005

^a^In adjusted analyses, both Cohort 1 and Cohort 2 adjusted for calendar year of the index date, sociodemographics, types of chronic pain, other comorbidities, frailty index, polypharmacy, healthcare utilization, and use of nonopioids during baseline as well as opioid measures (including dose and type) on the index date. Cohort 2 also adjusted for the opioid measures (including dose, type, and duration) during baseline.

^b^In adjusted analyses, both Cohort 1 and Cohort 2 adjusted for all variables mentioned in footnote a except for age group. Cohort 2 adjusted the above covariates as well as opioid measures (including dose, type, and duration) during baseline.

^c^In adjusted analyses, both Cohort 1 and Cohort 2 adjusted for all variables mentioned in footnote a except for sex. Cohort 2 adjusted the above covariates as well as opioid measures (including dose, type, and duration) during baseline.

^d^In adjusted analyses, both Cohort 1 and Cohort 2 adjusted for all variables mentioned in footnote a except for benzodiazepine use at baseline. Cohort 2 adjusted the above covariates as well as opioid measures (including dose, type, and duration) during baseline.

^e^In adjusted analyses, both Cohort 1 and Cohort 2 adjusted for all variables mentioned in footnote a except for prescription opioid dose on the index date. Cohort 2 adjusted the above covariates as well as opioid measures (including dose, type, and duration) during baseline.

aHR, adjusted hazard ratio; CI, confidence interval; HR, hazard ratio; MME, morphine milligram equivalent.

## Discussion

Using a nationally representative sample of older Medicare beneficiaries with CNCP, we found that the association between concurrent use of prescription gabapentinoids with opioids and risk for fall-related injury differed between new (Cohort 1) and prevalent (Cohort 2) opioid users. The risk for fall-related injury was 69% higher among older patients who initiated gabapentinoids with existing opioid therapy, whereas no risk difference was observed among older patients who initiated both gabapentinoids and opioids simultaneously, compared with their respective counterparts who used prescription opioids only. Sensitivity analyses in each cohort yielded similar findings. Notably, in Cohort 2 that compared patients who initiated gabapentinoids with existing opioid therapy versus those who continued using opioid only, an increased risk of fall-related injury was observed shortly (within 14 days) after the concurrent therapy initiation, and among patients who were aged ≥75, had no baseline use of benzodiazepine, and received opioid dose of ≥20 MME/day on the index date.

There has been a limited number of population-based studies that investigated the risk of fall-related injury associated with concurrent use of gabapentinoids and opioids. Only 1 study has been conducted using a self-controlled case series design among a mixed sample of young and older adults [25] and found a 40% higher rate of unintentional traumatic injury during the person time exposed to pregabalin–oxycodone versus oxycodone only; no association was detected between concurrent use of gabapentinoids with nonoxycodone opioids and injury risks. It is worth noting that the previously mentioned study did not differentiate concurrent use by whether patients had been on opioids before gabapentinoid initiation. We investigated the fall risks separately among concurrent users who initiated both drugs simultaneously (i.e., Cohort 1) and among concurrent users who initiated gabapentinoids with existing opioids (i.e., Cohort 2) and found increased risk exclusively in Cohort 2. The comparison of the 2 cohorts revealed that Cohort 2 seemed to have more types of chronic pain, and were at higher risk for OUD but lower risk for fall-related injury, confirming our initial rationale that the 2 cohorts represented distinct patient population and warranted separate investigations. Our study highlights the importance of considering the previous opioid use before gabapentinoid initiation when studying outcomes of concurrent gabapentinoid–opioid use [36,37].

The reasons behind discrepancies between Cohort 1 and Cohort 2 are unclear but may suggest existing opioid use (yes/no) as an effect modification of the association between concurrent use and fall risks. Although the mechanisms remain unknown, similar effect modification by existing opioid use has been noted in the interaction between opioids and certain CNS depressants [38,39]. In an animal model, pregabalin potentiated the opioid-induced drug-liking effects in mice with existing opioid levels but inhibited such effects in mice given opioids later [38]. In a population-based cohort study investigating the association between the use of muscle relaxants and risk for opioid overdose, an increased risk was observed among concurrent users who added muscle relaxants to existing opioids, but not among those who were opioid naive before initiating the concurrent therapy [39]. Alternatively, the observed discrepancies may be explained by a higher residual confounding in Cohort 2 than in Cohort 1. Our comparison of the 2 cohorts revealed that the differences in the unweighted baseline characteristics between concurrent users and opioid-only users were more pronounced in Cohort 2, implying more overall confounding in this cohort. Cohort 2 also seemed to have more types of chronic pain and were at higher risk for OUD, raising further concerns for residual confounding due to unmeasured factors such as pain severity, addiction, or substance use. For example, the clinical scenarios where gabapentinoids were initiated due to uncontrolled pain or early signs of OUD—either can independently increase fall risks [40,41]—would have biased our estimates toward a higher risk for fall in the concurrent users. Given its direction, this residual confounding is likely to explain our findings in Cohort 2 but not Cohort 1. Future studies, ideally using data containing information on indications and substance use records, are needed to determine whether or to what extent our findings in Cohort 2 were due to residual confounding.

Our study has several strengths, including its large size, emphasis on older adults, and investigation of gabapentinoid initiation among new and prevalent opioid users. Nevertheless, our results should be interpreted in light of several limitations. First, our study is subject to unmeasured confounders such as duration and severity of pain conditions, tolerance of opioid side effects, illicit opioid use, and clinical reasons (e.g., uncontrolled pain or early signs of opioid misuse [40,41]) for gabapentinoids initiation, all of which were not captured in Medicare claims data. Although we attempted to alleviate these confounding by adjusting for proxies, including baseline opioid duration and dose and diagnosis of OUD and alcohol/tobacco use disorder, there could be residual confounding. Second, Medicare Part D data only contains information on prescriptions reimbursed by Medicare. We were unable to capture medications reimbursed through other insurance mechanisms or purchased out of pocket or illicitly. There is also no information indicating whether drugs are used on a regular or as-needed basis. Lack of such information could lead to misclassification of prescription opioids and gabapentinoids treatment that likely biased our estimates toward the null. Finally, our findings are only generalizable to older adults insured by Medicare FFS programs.

Our study has important implications for public health, particularly given the recent surge of gabapentinoids use in the US [1,2]. Gabapentinoids have been recommended by AGS and CDC as adjuvants to be used with opioids, with an intention to reduce opioid consumption and thereby opioid-related adverse events. Recent safety concerns regarding the concurrent gabapentinoid–opioid use mainly derived from their additive CNS depressant effects. We investigated whether this concurrent use was associated with increased risk of fall—one of the most common and severe adverse events due to CNS depression among older adults [10]. The null results in Cohort 1 suggest initiating gabapentinoids with opioids was not associated with elevated risk of fall. However, the positive associations observed in Cohort 2, if confirmed by future studies, may suggest that clinicians should reevaluate gabapentinoids’ risk–benefit profiles before prescribing gabapentinoids to older adults who have been taking prescription opioids. If clinicians decide to add gabapentinoids to existing opioid treatment, they may need to heed the risk of fall-related injury during the first 14 days of concurrent use, and particularly among patients aged ≥75, without previous benzodiazepine use, and receiving opioids at ≥20 MME/day.

## Conclusions

In this sample of older Medicare beneficiaries with CNCP, we observed that initiating gabapentinoids and opioids simultaneously compared with initiating opioids only was not associated with risks for fall-related injury. However, addition of gabapentinoids to an existing opioid regimen was associated with increased fall risk. Mechanisms for the observed excess risk, whether pharmacological or because of channeling of combination therapy to high-risk patients, require further investigation. Clinicians should consider the risk–benefit of combination therapy when prescribing gabapentinoids concurrently with opioids.

## Supporting information

S1 Text The prospective analytical plan for study design and analysis. (DOCX)

S1 STROBE Checklist STROBE checklist. STROBE, Strengthening the Reporting of Observational Studies in Epidemiology.
(DOCX)

S1 Fig Kaplan–Meier survival curves for Cohort 1. (DOCX)

S2 Fig Kaplan–Meier survival curves for Cohort 2. (DOCX)

S1 Table ICD-9/10-CM codes for CNCP. CNCP, chronic noncancer pain.
(DOCX)

S2 Table List of covariates considered in the study. (DOCX)

S3 Table List of long-acting opioids. (DOCX)

S4 Table Complete baseline characteristics of Cohorts 1 and 2. (DOCX)

## Abbreviations

AGS: American Geriatric Society
aHR: adjusted hazard ratio
CDC: Center for Disease Control and Prevention
CI: confidence interval
CNCP: chronic noncancer pain
CNS: central nervous system
ED: emergency department
FFS: fee-for-service
IPTW: inverse probability of treatment weight
IQR: interquartile range
MME: morphine milligram equivalent
OUD: opioid use disorder
PS: propensity score
SMD: standardized mean difference
SNF: skilled nursing facility
STROBE: Strengthening the Reporting of Observational Studies in Epidemiology
