# Supplementary material for: Concurrent use of prescription gabapentinoids with opioids and risk for fall-related injury among older US Medicare beneficiaries with chronic noncancer pain: A population-based cohort study
Source: PLoS Med. 2022 Mar 1;19(3):e1003921. doi: 10.1371/journal.pmed.1003921 (PMC8887769; doi:10.1371/journal.pmed.1003921)
Supplement: S1 Text — (DOCX) [file pmed.1003921.s001.docx]

This is a pre-specified analysis plan. We detailed the steps of our analysis plan that were pre-specified before data were available for analysis as follows:

**Step 1. Identification of opioid initiators**

To improve computational efficiency of sample selection, we will construct our study sample in two steps: first we will assemble a source population of opioid initiators, and then we will sample the exposed and unexposed patients from this source population. The source population consist of older adults (aged ≥ 65 years) who were diagnosed with chronic noncancer pain (CNCP) and initiated a prescription opioid between 1/1/2011 and 12/31/2018. The list of opioids of interest is as follow:

| Buprenorphine^a^ | butorphanol | Codeine | Dihydrocodeine |
| --- | --- | --- | --- |
| Fentanyl | Hydrocodone | Hydromorphone | Levorphanol |
| Meperidine | Methadone | Morphine | Oxycodone |
| Oxymorphone | Pentazocine | Tapentadol | Tramadol |

*Notes.* Injectables are excluded because they are mostly for inpatient use. We also exclude buprenorphine indicated for opioid dependence and cough or cold medications containing opioids because they are unlikely to be used for CNCP treatment. Buprenorphine sublingual tablets, and buprenorphine-naloxone combinations are excluded as they are indicted for opioid dependence

Patients entered the source cohort when they initiated an opioid prescription, defined as filling an opioid prescription without receiving any opioids in the prior 6 months. During the 6 months before the opioid initiation date (i.e., pre-initiation period), we will require the patients to 1) be continuously enrolled in Medicare Parts A, B and D, and 2) have at least one diagnosis of CNCP (see S2 for diagnostic codes). During the 6-month pre-initiation period, we will exclude older adults who 1) had gabapentinoid exposure because prevalent gabapentinoid users may have different intrinsic risk of fall-related injury, 2) were covered by health maintenance organization (HMO) due to incomplete medical claims from these organizations, 3) had a cancer diagnosis or used palliative or hospice services because their opioid use patterns and risks for fall-related injury are different from who did not, and 4) had a fall-related injury because the 2012 Beers criteria recommended against the use of anticonvulsants among older adults with history of fall, thus patients with history of fall may be less likely to receive concurrent gabapentinoid-opioid (exposure) but more likely to have a subsequent fall (outcome). Patients will be eligible for being sampled from the opioid initiation day until they disenrolled from Medicare, died, discontinued opioids, experienced a fall-related injury, received a cancer diagnosis, used palliative or hospice services, or reached study end (12/31/2018), whichever came first. To account for nonadherence, we will extend the days’ supply of opioids by 20% when determining opioid discontinuation. Patients could reenter the cohort if they became eligible subsequently.

**Step 2. Sample the exposed and unexposed from the source population**

We will sample the exposed group and the unexposed group from the source cohort.

**Exposed groups.** The exposed group consists of opioid initiators who started gabapentinoids while continuously using prescription opioids. For an exposed patient, the first calendar day of concurrent use, i.e., having supplies of both prescription opioids and gabapentinoids, is defined as the index date. We will divide the exposed patient into two groups based on whether they initiated concurrent therapy on opioid initiation date (Cohort 1) or after opioid initiation date (Cohort 2) and assemble the unexposed group respectively for each. The rationale of separating these two is that they were expected to have different opioid needs and use patterns before gabapentinoid initiation, which may influence their baseline risks for fall.

**Unexposed groups.** Because the number of potential unexposed patients is considerably larger and they did not have an intrinsic index date to anchor, we will use incidence density sampling to obtain an unbiased sample of unexposed patients and manually assign each of them an index date. For each exposed patient, we will randomly select up to four unexposed patients from the source cohort who had the same cohort entry date as the exposed patient and will assign the unexposed patients same index date as the exposed patient. The unexposed patients are required to 1) be eligible for being sampled on the index date, and 2) did not have concurrent gabapentinoid-opioid use on the index date, or between opioid initiation date and the index date.

An unexposed patient may become exposed subsequently and vice versa. By the end of this sampling process, we should have two cohorts within which the exposed and unexposed patients should be 1:4 matched on both cohort entry date and index date. Given that opioid and gabapentinoid prescribing has changed significantly between 2011 and 2018, matching on these calendar dates increased the comparability of the clinical settings from which the exposed and unexposed were drawn.

**Step 3. Determine follow-up**

Patients will contribute person-time to the analysis from the index date until the first occurrence of the following: incident fall-related injury, discontinuation of opioids (allowing 20% days’ supply as the grace period), death, end of Medicare enrollment, day of cancer diagnosis, receiving hospice or palliative care, 30 days after index date, or end of the study period (i.e., 12/31/2018). For the exposed group, discontinuation of gabapentinoid exposure is also a censoring criterion. For the unexposed group, patients will also be censored when they initiated prescription gabapentinoids.

**Step 4. Ascertain outcome**

Our outcome of interest is incident fall-related injury that required outpatient or inpatient care. We will use a validated algorithm that includes *International Classification of Diseases, Ninth/Tenth Revision, Clinical Modification* (ICD-9/10-CM) and/or *current procedural terminology* codes for fall and injury. The algorithm has been validated against a composite standard of either an external cause of injury or confirmation by the Health and Retirement Study patients review. This algorithm has a sensitivity of 62.1%, a specificity of 98.8%, and a positive predictive value of 88.6%.

**Step 5. Measure baseline covariates**

We will measure covariates during a baseline period before each patient’s index date. The baseline is defined as the 6-month pre-initiation period plus the period between the opioid initiation date and index date (i.e., 6-month before index date in Cohort 1 and 6-month before opioid initiation plus the days between opioid initiation and index date in Cohort 2). Covariates that may be associated with concurrent gabapentinoid-opioid use and fall-related injury outcome are listed in S3. We will include sociodemographics, painful conditions, physical or neurologic/mental comorbidities that were identified as risk factors for fall, frailty, nonopioid medication use that increases risk of fall, and healthcare utilization variables. To better characterize chronic pain experienced by our study sample, we will group painful conditions into nine mutually exclusive categories: back pain, neck pain, gout, osteoarthritis, rheumatic disease, other musculoskeletal pain, neuropathy or neuralgia, fibromyalgia, and idiopathic pain. Since opioid use alone may increase the risk of fall-related injury, we adjusted for opioid use both before and on the index date. Pre-index opioid use was measured by days since opioid initiation, cumulative morphine milligram equivalent (MME; categorized based on quartiles), high dose opioid use (i.e., at least one day with MME≥90), and use of long-acting opioid (yes/no) before the index date. Index opioid use is measured as daily dose in MME and use of long-acting opioids on the index date. We will classify index opioid dose into four categories as: <20, 20-49, 50-89, and ≥90 MME. These cutoffs were commonly used by literature that investigate the risk of opioid-related adverse events.

**Step 6. Construct propensity score**

To balance baseline characteristics between the exposed and unexposed groups, we will use logistic regression models to construct a summary propensity score (PS)—the likelihood of receiving the concurrent therapy versus opioid monotherapy conditioned on baseline covariates. Then we will weigh the sample by inverse probability of treatment weights (IPTW). In IPTW, the exposed patients receive weights of the inverse of the estimated PS, whereas the unexposed patients receive weights equal to the inverse of 1 minus the estimated PS. To increase precision of the association analysis, we used stabilized IPTW by multiplying the previously defined weights by the marginal probability of receiving the corresponding treatment (i.e., concurrent therapy for exposed and monotherapy for unexposed). We will assess covariates' balance between the weighted cohorts using standardized mean differences (SMD), with an SMD of 0.1 or less indicating balance between groups.

**Step 7. Fit Cox proportional model**

Further analyses will be conducted based on the IPTW-adjusted pseudo-cohorts in which baseline characteristics of the exposed and unexposed were balanced. In each cohort, we will fit a Cox proportional hazard models with robust estimation to compare users of concurrent gabapentinoid-opioid versus users of opioid-only. We will calculate the incidence rate of fall-related injury for each group and report adjusted hazard ratios and associated 95% confidence intervals for each cohort. Schoenfeld Residuals will be calculated to test the proportional hazards assumptions.

**Step 8. Sensitivity analyses**

To evaluate the robustness of our main findings, we will conduct three sensitivity analyses. First, to address concerns about informative censoring, we will conduct an intention-to-treat analysis where patients are considered remaining in the initial treatment group, regardless of discontinuation or switching during follow-up. Second, to account for changes in co-medication use during follow-up that may influence the risk of falls, we will model daily MME, use of long-acting opioids, and use of other medications associated with fall risks as time-varying covariates. Third, to reduce the potential effect of previous fractures or osteoporosis during the baseline period on our estimate, we will exclude patients with a diagnosis of these two conditions and those who filled prescriptions for anti-osteoporosis medications during the baseline period.

**Step 9. Subgroup analyses**

To assess whether the association varied by treatment patterns, we will conduct several subgroup analyses in each cohort according to individual gabapentinoid agents (i.e., gabapentin or pregabalin), duration of concurrent gabapentinoid-opioid use, and starting dose of gabapentinoid treatment. We will consider high starting dose as >300mg/day for gabapentin or >150 mg/day for pregabalin, the recommended starting dose per US Food and Drug Administration approved label. For all subgroup analyses in which a subset of samples was used, we will recalculate the IPTWs and reassessed the balance of the covariates.
