# Supplementary material for: Concurrent use of prescription gabapentinoids with opioids and risk for fall-related injury among older US Medicare beneficiaries with chronic noncancer pain: A population-based cohort study
Source: PLoS Med. 2022 Mar 1;19(3):e1003921. doi: 10.1371/journal.pmed.1003921 (PMC8887769; doi:10.1371/journal.pmed.1003921)

S2 Fig: Kaplan-Meier Survival Curves for Cohort 2

Figs (a) and (b) depict the unadjusted and adjusted Kaplan-Meier (KM) curves for Cohort 1, respectively. In both figures, X-axis indicates follow-up days, and Y-axis indicates the proportion of the study sample who were event-free. The blue solid line represents the opioid-only users (i.e., concurrent use=0), and the red dotted line represents the concurrent users (i.e., concurrent use=1). The blue shaded regions indicate the 95% Hall-Wellner confidence bands of the KM curve for the opioid-only users, and the red shade indicate the 95% Hall-Wellner confidence bands of the KM curve for the concurrent users. In Fig (b), the KM curve was adjusted for calendar year of the index date, sociodemographics, types of chronic pain, other comorbidities, frailty index, polypharmacy, healthcare utilization, and use of non-opioids during baseline, opioid measures (including dose and type) on the index date, and the opioid measures (including dose, type, and duration) during baseline.


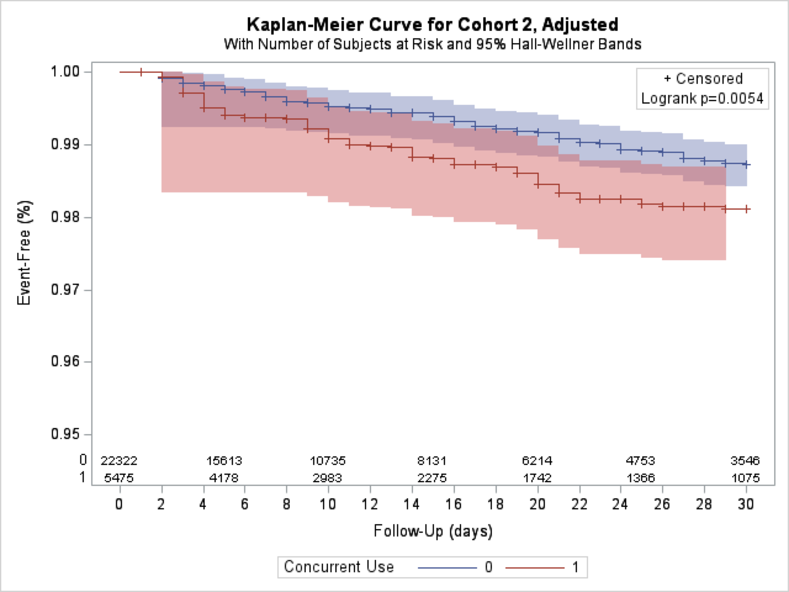


**(b)**

**(a)**


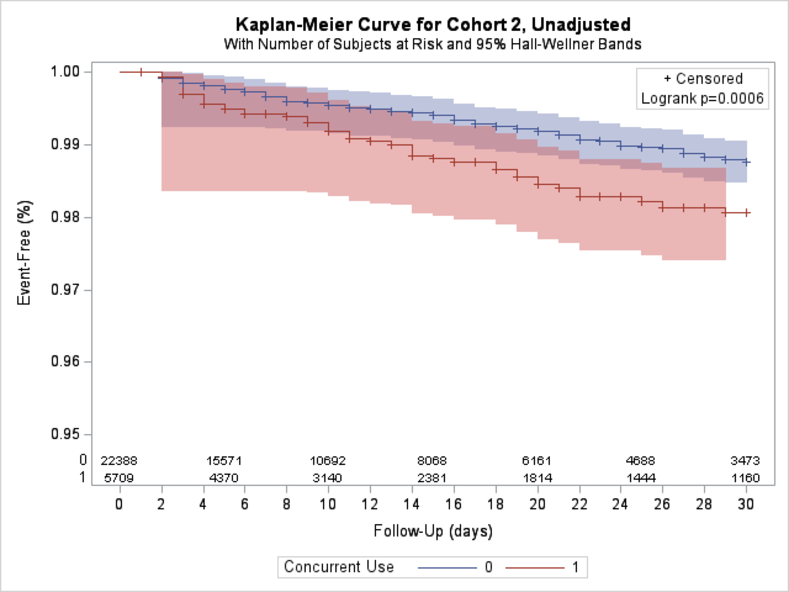

Supplement: S2 Fig — (DOCX) [file pmed.1003921.s004.docx]
