# Supplementary material for: Concurrent use of prescription gabapentinoids with opioids and risk for fall-related injury among older US Medicare beneficiaries with chronic noncancer pain: A population-based cohort study
Source: PLoS Med. 2022 Mar 1;19(3):e1003921. doi: 10.1371/journal.pmed.1003921 (PMC8887769; doi:10.1371/journal.pmed.1003921)
Supplement: S1 Table — CNCP, chronic noncancer pain. (DOCX) [file pmed.1003921.s005.docx]

S1 Table. *ICD-9/10-CM* Codes for Chronic Noncancer Pain

| Chronic pain conditions | ICD-9-CM | ICD-10-CM |
| --- | --- | --- |
| Back pain | 720.x, 721.2,721.3, 721.5-721.8, 721.90, 722.x (except 722.4, 722.7x, 722.81, 722.91), 724.x (except 724.03, 724.3, 724.4) | M08.1, M25.78, M43.20, M43.24-M43.28, M43.8X9, M45.x (except M45.1-M45.3), M46.0x (except M46.01-M46.03), M46.4x(except M46.41-M46.43), M46.5x(except M46.51-M46.53), M46.8x(except M46.81-M46.83), M46.9x (except M46.91-M46.93), M47.2x (except M47.21-M47.23), M47.8x (except M47.811-M47.813, M47.891-M47.893),M47.9, M48.0x (except M48.01-M48.03, M48.062), M48.1x(except M48.11-M48.13), M48.2x(except M48.21-M48.23), M48.3x(except M48.31-M48.33), M48.8Xx(except M48.8X1-M48.8X3), M49.8x (except M49.81-M49.83), M51.x (except M51.0x), M53.2X7, M53.2X8, M53.3, M53.8x(except M53.81-M53.83), M53.9, M54.0x (except M54.01-M54.03), M54.5-M54.9,M62.830, M96.1, M99.22-M99.25, M99.32-M99.35, M99.42-M99.45, M99.52-M99.55, M99.62-M99.65, M99.72-M99.75 |
| Neck pain | 721.0,722.0,722.4,722.81,722.91,723.0-723.3,723.5-723.9 | M43.6, M45.1-M45.3, M46.01-M46.03, M46.41-M46.43, M46.51-M46.53, M46.81-M46.83, M46.91-M46.93, M47.21-47.23, M47.811-M47.813, M47.891-M47.893, M48.01-M48.03, M48.11-M48.13, M48.21-M48.23, M48.31-M48.33, M48.8X1-M48.8X3,M49.81-M49.83, M50.x (except M50.0x), M53.0, M53.1, M53.81-M53.83, M54.01-M54.03, M54.2, M67.88, M96.1, M99.21, M99.31, M99.41, M99.51, M99.61, M99.71 |
| Osteoarthritis | 715.x | M15-M19 |
| Rheumatologic autoimmune disease | 710.0-710.4, 714.0-714.3, 714.81, 719.3, 725.x, 729.0 | M05.x, M06.x, M08.x, M12.3x, M12.4x, M32.x, M33.x, M34.x, M35.0x, M35.3, M79.0 |
| Gout and crystal arthropathies | 274.x, 712.x | M10.x, M11.x, M1A.x |
| Other joint pain | 711.x, 713.x, 714.4, 714.8x, 714.9, 716.x, 717.x, 718.x, 719.x (except 719.3) | A18.01, A18.02, A52.16, E08.610, E09.610, E10.610, E11.610, E13.610, M00.x-M02.x, M07.6x, M12.x (except M12.3x, M12.4x), M13.x-M14.x, M22.x-M25.x (except M24.2x, M25.78), M35.2, M36.1-M36.4, M53.2Xx (except M53.2X7, M53.2X8), R26.2, R29.4 |
| Other musculoskeletal pain | 710.5-710.9, 726.x, 727.x, 728.x, 729.3x-729.9x | D48.1, M04.x (except M04.2-M04.9), M20.10, M21.61x, M21.62x, M24.2x, M35.1, M35.4-M35.9, M36.8, M47.30, M60.0x-M60.2x, M61.x-M62.x (except M62.830), M65.x-M67.x (except M67.30, M67.88), M70.x-M72.x, M75.x-M77.x, M79.3-M79.9, M79.Ax, R25.2, R29.898 |
| Neuropathy or neuralgia | 053.1x, 249.6x, 250.6x, 337.x, 338.0x, 350.x, 351.x,353.x, 354.x,355.x,356.x (except 356.3), 357.x (except 357.0,357.5-357.7), 721.1, 721.4x, 721.91, 722.7x, 723.4, 724.03, 724.3, 724.4, 729.2 | A52.15, B02.x,  E08.4x,E08.610, E09.4x, E10.4x, E11.4x, E13.4x, G13.0, G13.1, G50.x, G51.x, G52.1, G54.x, G56.x, G57.x, G58.7, G60.x (except G60.1), G61.8x, G61.9, G62.81, G63, G65.x, G89.0, G90.x (except G90.1-G90.3), G99.0, M05.5x, M47.01x, M47.02x, M47.1x, M48.062, M50.0x, M51.0x, M54.1x, M54.3x, M54.4x, M79.2 |
| Idiopathic pain | 338.2x, 338.4, 780.96 | G89.2x, G89.4, R52 |
