# Supplementary material for: Concurrent use of prescription gabapentinoids with opioids and risk for fall-related injury among older US Medicare beneficiaries with chronic noncancer pain: A population-based cohort study
Source: PLoS Med. 2022 Mar 1;19(3):e1003921. doi: 10.1371/journal.pmed.1003921 (PMC8887769; doi:10.1371/journal.pmed.1003921)
Supplement: S2 Table — (DOCX) [file pmed.1003921.s006.docx]

S2 Table. List of Covariates Considered in the Study

| Categories | Covariates |
| --- | --- |
| Sociodemographics | Age at baseline, sex, low-income subsidy, race/ethnicity, metropolitan residence, geographic region, calendar year |
|  |  |
| Painful conditions | Back pain, neck pain, gout, joint pain, osteoarthritis, rheumatic disease, other musculoskeletal pain, neuralgia, fibromyalgia, abdominal pain, migraine, unspecified pain |
|  |  |
| Physical comorbidities | Acute renal failure, chronic kidney disease, congestive heart failure, diabetes, fracture, hearing impairment, hyperlipidemia, hypertension, liver disease, mobility impairment, myocardial infarction, obesity, osteoporosis, stroke, gastrointestinal disease, urinary incontinence |
|  |  |
| Neurologic/Mental comorbidities | Alzheimer’s disease and related dementia (ADRD), anxiety, depression, epilepsy, Parkinson’s disease and other neurodegenerative disease, alcohol/tobacco use disorder, opioid use disorder |
|  |  |
| Frailty | Frailty index (FI) categorized as non-frail (FI<0.15), pre-frail (0.15≤FI<0.25), mildly frail (0.25≤FI<0.35), and moderate-to-severely frail (FI≥0.35). |
|  |  |
| Healthcare utilization | Polypharmacy, any inpatient stay (yes/no), any ED visits (yes/no), any SNF stays (yes/no), any bone mineral density testing |
|  |  |
| Baseline nonopioid medication use | Anticonvulsants, TCA, SSRI, SNRI, antipsychotics, benzodiazepines, non-benzodiazepines sedatives/hypnotics, muscle relaxants, antihistamine, angiotensin II receptor antagonists, ACE inhibitors, beta-blockers, loop diuretics, thiazide diuretics, calcium channel blockers, antiosteoporosis, oral steroids |
|  |  |
| Index opioid prescription | Daily MME, use of long-acting opioid |
|  |  |
| Baseline opioid use^a^ | Opioid duration (in days), average MME, maximum MME, use of long-acting opioids (including long-acting or extended release form of buprenorphine, bentanyl, hydrocodone, levorphanol, methadone, morphine, oxycodone, oxymorphone, tapentadol, and tramadol) |

ED=Emergency department; SNF=Skilled nursing facility; ACE=Angiotensin-converting enzyme; TCAs=Tricyclic antidepressant; SNRIs=Serotonin and norepinephrine reuptake inhibitors; SSRIs=Selective serotonin reuptake inhibitors; MME=Morphine milligram equivalent.

^a^ Only adjusted in Cohort 2.
