# Supplementary material for: Concurrent use of prescription gabapentinoids with opioids and risk for fall-related injury among older US Medicare beneficiaries with chronic noncancer pain: A population-based cohort study
Source: PLoS Med. 2022 Mar 1;19(3):e1003921. doi: 10.1371/journal.pmed.1003921 (PMC8887769; doi:10.1371/journal.pmed.1003921)
Supplement: S3 Table — (DOCX) [file pmed.1003921.s007.docx]

S3 Table. List of long-acting opioids.

| **Generic Name** | **Dosage Form** |
| --- | --- |
| Buprenorphine | Patch, transdermal |
| Buprenorphine | Film, medicated (Each) |
| Fentanyl | Patch, transdermal |
| Fentanyl | Spray, non-aerosol (Each) |
| Fentanyl | Aerosol, spray with pump (Each) |
| Hydrocodone bitartrate | Capsule, extended release |
| Hydrocodone bitartrate | Tablet, extended release |
| Hydromorphone hcl | Tablet, extended release |
| Levorphanol tartrate | Tablet |
| Methadone | Concentrate, oral |
| Methadone | Solution, oral |
| Methadone | Tablet |
| Morphine sulfate | Tablet, extended release |
| Morphine sulfate/naltrexone | Capsule, extended release |
| Oxycodone | Tablet, extended release |
| Oxymorphone | Tablet, extended release |
| Tapentadol | Tablet, extended release |
| Tramadol | Tablet, extended release |
