# Supplementary material for: Concurrent use of prescription gabapentinoids with opioids and risk for fall-related injury among older US Medicare beneficiaries with chronic noncancer pain: A population-based cohort study
Source: PLoS Med. 2022 Mar 1;19(3):e1003921. doi: 10.1371/journal.pmed.1003921 (PMC8887769; doi:10.1371/journal.pmed.1003921)
Supplement: S4 Table — (DOCX) [file pmed.1003921.s008.docx]

S4 Table. Complete baseline characteristics of older adults with chronic noncancer pain at baseline according to treatment with concomitant use of gabapentinoids and opioids or opioids only.

| Baseline Characteristics | Cohort 1, % | | | | | | |  | Cohort 2, % | | | | | | |
| --- | --- | --- | --- | --- | --- | --- | --- | --- | --- | --- | --- | --- | --- | --- | --- |
|  | Before Propensity Score Weighting | | |  | After Propensity Score Weighting | | |  | Before Propensity Score Weighting | | |  | After Propensity Score Weighting | | |
|  | GABA-OPI  (n=6,733) | OPI-Only  (n=27,092) | SMD |  | GABA-OPI  (n=6,349) | OPI-Only  (n=27,156) | SMD |  | GABA-OPI  (n=5,709) | OPI-Only  (n=22,388) | SMD |  | GABA-OPI  (n=5,475) | OPI-Only  (n=22,322) | SMD |
| Year |  |  |  |  |  |  |  |  |  |  |  |  |  |  |  |
| 2011 | 4.5 | 4.5 | 0.00 |  | 4.7 | 4.5 | 0.07 |  | 4.5 | 4.6 | 0.00 |  | 4.7 | 4.6 | 0.04 |
| 2012 | 9.1 | 9.1 |  |  | 9.6 | 9.1 |  |  | 9.9 | 9.9 |  |  | 10.2 | 9.9 |  |
| 2013 | 10.7 | 10.7 |  |  | 10.8 | 10.7 |  |  | 12.8 | 12.8 |  |  | 13.0 | 12.8 |  |
| 2014 | 12.9 | 12.9 |  |  | 12.9 | 12.9 |  |  | 13.2 | 13.2 |  |  | 13.2 | 13.2 |  |
| 2015 | 12.4 | 12.4 |  |  | 12.5 | 12.4 |  |  | 13.3 | 13.3 |  |  | 13.5 | 13.4 |  |
| 2016 | 15.9 | 15.9 |  |  | 15.2 | 15.8 |  |  | 16.8 | 16.8 |  |  | 16.2 | 16.8 |  |
| 2017 | 16.9 | 16.9 |  |  | 16.4 | 16.9 |  |  | 16.1 | 16.1 |  |  | 15.6 | 16.1 |  |
| 2018 | 17.6 | 17.6 |  |  | 17.9 | 17.6 |  |  | 13.2 | 13.3 |  |  | 13.6 | 13.2 |  |
| Age, mean±SD, y | 75.0±7.0 | 75.7±7.6 | -0.10 |  | 75.4±7.0 | 75.6±7.5 | -0.03 |  | 75.7±7.3 | 76.5±7.8 | -0.11 |  | 76.3±7.4 | 76.4±7.8 | -0.01 |
| Female | 66.0 | 64.1 | 0.00 |  | 65.4 | 64.5 | 0.01 |  | 67.9 | 67.5 | 0.01 |  | 68.1 | 67.6 | 0.01 |
| Race/ethnicity |  |  | 0.16 |  |  |  | 0.04 |  |  |  | 0.07 |  |  |  | 0.09 |
| Non-Hispanic White | 77.4 | 81.8 |  |  | 80.2 | 80.9 |  |  | 80.7 | 79.2 |  |  | 79.6 | 79.5 |  |
| Black | 8.1 | 8.0 |  |  | 8.2 | 8.0 |  |  | 7.0 | 9.1 |  |  | 8.4 | 8.7 |  |
| Hispanic | 8.2 | 5.9 |  |  | 6.6 | 6.3 |  |  | 7.2 | 7.1 |  |  | 7.4 | 7.1 |  |
| Asian/Pacific Islander | 4.2 | 2.3 |  |  | 3.0 | 2.7 |  |  | 3.3 | 2.8 |  |  | 3.0 | 2.9 |  |
| Others | 2.1 | 2.0 |  |  | 2.0 | 2.0 |  |  | 1.7 | 1.8 |  |  | 1.6 | 1.8 |  |
| Non-metropolitan residence | 78.5 | 77.2 | 0.03 |  | 77.4 | 77.4 | 0.00 |  | 77.4 | 77.0 | 0.01 |  | 76.6 | 77.1 | -0.01 |
| Low-income subsidy | 26.0 | 22.7 | 0.08 |  | 24.4 | 23.4 | 0.02 |  | 25.0 | 28.6 | -0.08 |  | 28.5 | 27.9 | 0.01 |
| US Region^a^ |  |  | 0.06 |  |  |  | 0.03 |  |  |  | 0.05 |  |  |  | 0.03 |
| Northeast | 16.3 | 16.5 |  |  | 15.7 | 16.3 |  |  | 17.3 | 15.7 |  |  | 16.3 | 16.1 |  |
| Midwest | 22.2 | 23.5 |  |  | 23.1 | 23.2 |  |  | 22.0 | 23.5 |  |  | 22.3 | 23.2 |  |
| South | 42.5 | 41.6 |  |  | 42.6 | 41.9 |  |  | 41.5 | 42.3 |  |  | 42.3 | 42.2 |  |
| West | 19.0 | 18.4 |  |  | 18.6 | 18.6 |  |  | 19.2 | 18.4 |  |  | 19.2 | 18.6 |  |
| Chronic Pain |  |  |  |  |  |  |  |  |  |  |  |  |  |  |  |
| Back pain | 47.1 | 36.1 | 0.22 |  | 41.6 | 38.5 | 0.06 |  | 62.1 | 42.9 | 0.39 |  | 50.5 | 46.8 | 0.07 |
| Neck pain | 16.8 | 12.4 | 0.13 |  | 14.9 | 13.4 | 0.04 |  | 20.7 | 13.8 | 0.18 |  | 17.2 | 15.3 | 0.05 |
| Osteoarthritis | 48.1 | 41.4 | 0.14 |  | 44.5 | 42.8 | 0.03 |  | 42.0 | 47.5 | -0.11 |  | 46.4 | 46.5 | 0.00 |
| Rheumatic disease | 5.5 | 5.4 | 0.00 |  | 5.7 | 5.5 | 0.01 |  | 6.8 | 6.8 | 0.00 |  | 7.5 | 6.9 | 0.02 |
| Gout | 5.4 | 6.3 | -0.04 |  | 5.8 | 6.1 | -0.01 |  | 5.5 | 6.5 | -0.04 |  | 6.1 | 6.3 | -0.01 |
| Other joint pain | 50.4 | 46.0 | 0.09 |  | 48.1 | 47.0 | 0.02 |  | 51.9 | 51.0 | 0.02 |  | 52.5 | 51.3 | 0.02 |
| Other musculoskeletal pain | 46.5 | 46.6 | 0.00 |  | 47.3 | 46.6 | 0.01 |  | 54.4 | 48.5 | 0.12 |  | 52.5 | 49.9 | 0.05 |
| Neuralgia | 53.7 | 25.7 | 0.60 |  | 34.9 | 31.6 | 0.07 |  | 66.2 | 28.7 | 0.81 |  | 38.9 | 36.2 | 0.06 |
| Fibromyalgia | 7.7 | 5.6 | 0.08 |  | 7.0 | 6.1 | 0.03 |  | 10.2 | 6.6 | 0.13 |  | 8.6 | 7.4 | 0.04 |
| Abdominal pain | 14.8 | 19.4 | -0.12 |  | 17.8 | 18.5 | -0.02 |  | 19.4 | 18.0 | 0.04 |  | 18.7 | 18.3 | 0.01 |
| Migraine | 8.3 | 7.9 | 0.02 |  | 8.4 | 8.0 | 0.02 |  | 10.7 | 8.3 | 0.08 |  | 9.4 | 8.8 | 0.02 |
| Unspecified pain | 12.4 | 7.6 | 0.16 |  | 9.6 | 8.6 | 0.03 |  | 16.3 | 10.1 | 0.18 |  | 13.2 | 11.5 | 0.05 |
| Physical comorbidities |  |  |  |  |  |  |  |  |  |  |  |  |  |  |  |
| Acute renal failure | 5.2 | 4.5 | 0.03 |  | 4.9 | 4.7 | 0.01 |  | 4.8 | 5.4 | -0.03 |  | 5.3 | 5.2 | 0.00 |
| Chronic kidney disease | 20.0 | 20.2 | 0.00 |  | 20.2 | 20.1 | 0.01 |  | 20.8 | 21.7 | 0.00 |  | 22.2 | 21.5 | 0.02 |
| Congestive heart disease | 11.1 | 11.1 | 0.00 |  | 11.6 | 11.1 | 0.00 |  | 13.2 | 13.1 | -0.02 |  | 14.0 | 13.2 | 0.02 |
| Diabetes | 36.0 | 31.0 | 0.11 |  | 33.9 | 32.1 | 0.04 |  | 36.4 | 33.9 | 0.05 |  | 35.8 | 34.6 | 0.02 |
| Fracture | 4.2 | 5.5 | -0.06 |  | 5.2 | 5.2 | 0.00 |  | 6.4 | 6.3 | 0.00 |  | 6.6 | 6.4 | 0.01 |
| Hearing impairment | 5.1 | 5.1 | 0.00 |  | 5.0 | 5.0 | 0.00 |  | 5.8 | 5.5 | 0.01 |  | 6.1 | 5.6 | 0.02 |
| Hyperlipidemia | 66.5 | 63.4 | 0.07 |  | 64.8 | 64.0 | 0.02 |  | 68.3 | 65.1 | 0.07 |  | 66.4 | 65.7 | 0.02 |
| Hypertension | 77.2 | 74.1 | 0.07 |  | 75.7 | 74.8 | 0.02 |  | 78.5 | 77.9 | 0.01 |  | 78.3 | 78.1 | 0.00 |
| Liver disease | 3.7 | 4.2 | -0.02 |  | 4.1 | 4.1 | 0.00 |  | 4.3 | 4.0 | 0.01 |  | 4.1 | 4.1 | 0.00 |
| Mobility impairment | 2.7 | 1.8 | 0.06 |  | 2.1 | 1.9 | 0.01 |  | 2.5 | 2.2 | 0.02 |  | 2.4 | 2.3 | 0.01 |
| Myocardial Infarction | 0.9 | 1.1 | -0.01 |  | 1.1 | 1.0 | 0.00 |  | 1.6 | 1.2 | 0.03 |  | 1.5 | 1.3 | 0.01 |
| Obesity | 17.6 | 13.9 | 0.10 |  | 15.7 | 14.7 | 0.03 |  | 14.6 | 14.1 | 0.01 |  | 14.9 | 14.3 | 0.02 |
| Osteoporosis | 11.3 | 10.2 | 0.03 |  | 10.7 | 10.4 | 0.01 |  | 13.2 | 12.5 | 0.02 |  | 13.7 | 12.7 | 0.03 |
| Stroke | 5.4 | 5.3 | 0.01 |  | 5.5 | 5.3 | 0.01 |  | 6.9 | 6.3 | 0.02 |  | 6.8 | 6.5 | 0.01 |
| Gastrointestinal disease | 21.7 | 22.6 | -0.02 |  | 22.3 | 22.3 | 0.00 |  | 25.9 | 24.3 | 0.04 |  | 24.6 | 24.6 | 0.00 |
| Urinary incontinence | 15.6 | 14.9 | 0.02 |  | 15.0 | 15.0 | 0.00 |  | 17.5 | 17.3 | 0.01 |  | 17.9 | 17.4 | 0.01 |
| Neurologic/Mental comorbidities |  |  |  |  |  |  |  |  |  |  |  |  |  |  |  |
| ADRD | 4.1 | 5.5 | -0.07 |  | 5.0 | 5.2 | -0.01 |  | 5.9 | 7.1 | -0.05 |  | 7.0 | 6.9 | 0.01 |
| Anxiety | 13.0 | 12.5 | 0.01 |  | 13.0 | 12.6 | 0.01 |  | 14.7 | 13.2 | 0.04 |  | 14.1 | 13.6 | 0.01 |
| Depression | 14.3 | 13.4 | 0.03 |  | 14.2 | 13.6 | 0.02 |  | 16.6 | 15.4 | 0.03 |  | 16.9 | 15.7 | 0.03 |
| Epilepsy | 1.2 | 1.3 | -0.01 |  | 1.1 | 1.2 | -0.01 |  | 1.2 | 1.4 | -0.01 |  | 1.5 | 1.4 | 0.01 |
| Neurodegenerative disease | 6.8 | 5.8 | 0.04 |  | 6.2 | 6.0 | 0.01 |  | 8.4 | 6.9 | 0.06 |  | 8.3 | 7.3 | 0.04 |
| Alcohol/Tobacco use disorder | 9.0 | 7.8 | 0.04 |  | 8.1 | 8.0 | 0.00 |  | 10.8 | 8.4 | 0.08 |  | 9.6 | 8.9 | 0.03 |
| Opioid use disorder | 0.9 | 0.3 | 0.08 |  | 0.5 | 0.4 | 0.01 |  | 1.8 | 0.6 | 0.12 |  | 1.0 | 0.8 | 0.02 |
| Frailty index, mean±SD | 0.2±0.1 | 0.2±0.1 | 0.09 |  | 0.2±0.1 | 0.2±0.1 | 0.04 |  | 0.2±0.1 | 0.2±0.1 | 0.09 |  | 0.2±0.1 | 0.2±0.1 | 0.05 |
| Healthcare utilization |  |  |  |  |  |  |  |  |  |  |  |  |  |  |  |
| Polypharmacy | 92.2 | 84.3 | 0.24 |  | 88.3 | 85.9 | 0.07 |  | 83.3 | 79.3 | 0.10 |  | 81.6 | 80.1 | 0.04 |
| Hospitalization | 30.8 | 17.5 | 0.31 |  | 22.0 | 20.4 | 0.04 |  | 20.3 | 19.0 | 0.03 |  | 20.6 | 19.3 | 0.03 |
| ED visit | 26.3 | 28.8 | -0.06 |  | 27.8 | 28.3 | -0.01 |  | 39.0 | 27.2 | 0.25 |  | 30.4 | 29.4 | 0.02 |
| SNF services | 5.3 | 2.9 | 0.12 |  | 3.8 | 3.4 | 0.02 |  | 4.7 | 4.1 | 0.03 |  | 4.6 | 4.3 | 0.02 |
| Bone mineral testing | 5.9 | 5.6 | 0.01 |  | 5.7 | 5.6 | 0.00 |  | 6.8 | 6.1 | 0.03 |  | 6.5 | 6.3 | 0.01 |
| Baseline medication use |  |  |  |  |  |  |  |  |  |  |  |  |  |  |  |
| Anticonvulsants | 2.6 | 3.5 | -0.05 |  | 3.0 | 3.3 | -0.02 |  | 3.7 | 3.9 | -0.01 |  | 4.2 | 3.9 | 0.01 |
| TCA | 3.3 | 2.6 | 0.04 |  | 2.9 | 2.7 | 0.01 |  | 3.5 | 3.2 | 0.02 |  | 3.4 | 3.2 | 0.01 |
| SSRI | 16.2 | 16.4 | -0.01 |  | 17.0 | 16.4 | 0.02 |  | 18.4 | 17.8 | 0.02 |  | 18.4 | 17.9 | 0.01 |
| SNRI | 4.4 | 4.4 | 0.00 |  | 4.8 | 4.4 | 0.02 |  | 6.3 | 5.0 | 0.05 |  | 5.9 | 5.4 | 0.02 |
| Antipsychotics | 2.3 | 3.4 | -0.06 |  | 3.0 | 3.2 | -0.01 |  | 4.1 | 4.5 | -0.02 |  | 4.9 | 4.5 | 0.02 |
| Benzodiazepines | 12.6 | 13.8 | -0.03 |  | 13.7 | 13.6 | 0.00 |  | 16.9 | 15.3 | 0.04 |  | 15.9 | 15.7 | 0.01 |
| Nonbenzodiazepines^b^ | 6.8 | 6.3 | 0.02 |  | 6.5 | 6.4 | 0.01 |  | 7.1 | 6.8 | 0.01 |  | 6.8 | 6.8 | 0.00 |
| Muscle relaxants | 12.8 | 9.3 | 0.11 |  | 11.1 | 10.1 | 0.03 |  | 18.7 | 11.0 | 0.22 |  | 13.3 | 12.5 | 0.02 |
| Antihistamine | 4.5 | 4.9 | -0.02 |  | 4.9 | 4.8 | 0.00 |  | 5.6 | 5.2 | 0.02 |  | 5.9 | 5.3 | 0.02 |
| Angiotensin II receptor blockers | 25.3 | 22.7 | 0.06 |  | 24.1 | 23.3 | 0.02 |  | 24.2 | 23.9 | 0.01 |  | 23.5 | 23.9 | -0.01 |
| ACE inhibitor | 28.6 | 28.5 | 0.00 |  | 28.9 | 28.6 | 0.01 |  | 28.9 | 29.5 | -0.01 |  | 29.5 | 29.4 | 0.00 |
| Beta-blocker | 39.5 | 40.4 | -0.02 |  | 40.9 | 40.3 | 0.01 |  | 42.8 | 43.0 | 0.00 |  | 43.8 | 43.1 | 0.01 |
| Loop diuretics | 14.2 | 15.3 | -0.03 |  | 15.2 | 15.1 | 0.01 |  | 17.0 | 17.9 | -0.02 |  | 18.6 | 17.9 | 0.02 |
| Thiazide diuretics | 25.1 | 22.3 | 0.07 |  | 23.6 | 22.9 | 0.01 |  | 23.8 | 23.2 | 0.01 |  | 23.3 | 23.3 | 0.00 |
| Calcium channel blocker | 28.2 | 28.1 | 0.00 |  | 28.3 | 28.2 | 0.00 |  | 29.2 | 30.2 | -0.02 |  | 30.0 | 30.0 | 0.00 |
| Antiosteoporosis | 5.8 | 5.4 | 0.02 |  | 5.5 | 5.4 | 0.00 |  | 7.1 | 6.5 | 0.02 |  | 6.6 | 6.5 | 0.00 |
| Oral steroids | 30.1 | 27.4 | 0.06 |  | 29.3 | 28.0 | 0.03 |  | 42.9 | 27.8 | 0.32 |  | 32.2 | 30.8 | 0.03 |
| Index opioid prescription | |  |  |  |  |  |  |  |  |  |  |  |  |  |  |
| MME group |  |  | 0.25 |  |  |  | 0.05 |  |  |  | 0.29 |  |  |  | 0.05 |
| <20 | 33.6 | 28.9 |  |  | 30.1 | 29.7 |  |  | 34.6 | 45.4 |  |  | 43.3 | 43.4 |  |
| 20-49 | 46.1 | 55.8 |  |  | 53.2 | 53.8 |  |  | 45.2 | 44.0 |  |  | 43.4 | 44.3 |  |
| 50-89 | 13.0 | 12.2 |  |  | 12.2 | 12.4 |  |  | 13.5 | 7.7 |  |  | 9.1 | 8.7 |  |
| ≥90 | 7.3 | 3.1 |  |  | 4.5 | 4.1 |  |  | 6.7 | 2.9 |  |  | 4.2 | 3.6 |  |
| Use of long-acting opioids | 2.3 | 0.7 | 0.13 |  | 1.2 | 1.0 | 0.01 |  | 3.6 | 2.0 | 0.10 |  | 3.1 | 2.4 | 0.04 |
| Baseline opioid use |  |  |  |  |  |  |  |  |  |  |  |  |  |  |  |
| Days, mean±SD | – | – | – |  | – | – | – |  | 17.8±34.5 | 15.2±24.5 | 0.09 |  | 16.6±29.7 | 15.8±27.5 | 0.03 |
| Average MME |  |  |  |  |  |  |  |  |  |  | 0.12 |  |  |  | 0.00 |
| <20 | – | – | – |  | – | – | – |  | 41.2 | 46.5 |  |  | 44.6 | 45.4 |  |
| 20-49 | – | – | – |  | – | – | – |  | 48.0 | 43.6 |  |  | 44.5 | 44.5 |  |
| 50-89 | – | – | – |  | – | – | – |  | 8.3 | 7.5 |  |  | 8.1 | 7.7 |  |
| ≥90 | – | – | – |  | – | – | – |  | 2.4 | 2.3 |  |  | 2.8 | 2.4 |  |
| Max MME |  |  |  |  |  |  |  |  |  |  | 0.16 |  |  |  | 0.03 |
| <20 | – | – | – |  | – | – | – |  | 35.6 | 42.3 |  |  | 39.7 | 40.9 |  |
| 20-49 | – | – | – |  | – | – | – |  | 45.5 | 44.8 |  |  | 44.6 | 44.9 |  |
| 50-89 | – | – | – |  | – | – | – |  | 13.1 | 9.0 |  |  | 10.6 | 9.9 |  |
| ≥90 | – | – | – |  | – | – | – |  | 5.8 | 3.9 |  |  | 5.1 | 4.3 |  |
| Use of long-acting opioids | – | – | – |  | – | – | – |  | 3.4 | 2.0 | 0.09 |  | 3.1 | 2.4 | 0.04 |

GABA-OPI=Gabapentinoid-Opioid; OPI-Only=Opioid-Only; SMD=Standardized mean difference; ED=Emergency department; SNF=Skilled nursing facility; ACE=Angiotensin-converting enzyme; TCAs=Tricyclic antidepressant; SNRIs=Serotonin and norepinephrine reuptake inhibitors; SSRIs=Selective serotonin reuptake inhibitors; MME=Morphine milligram equivalent.

^a^ US region was defined by the state code of a beneficiary’s residence and classified per the U.S. census bureau.

^b^ Nonbenzodiazepines included eszopiclone, zaleplon, and zolpidem.
